# Long-Term Outcomes of Allergic Bronchopulmonary Aspergillosis and *Aspergillus* Colonization in Children and Adolescents with Cystic Fibrosis

**DOI:** 10.3390/jof10090599

**Published:** 2024-08-24

**Authors:** Emily Chesshyre, Fiona C. Warren, Angela C. Shore, Jane C. Davies, Darius Armstrong-James, Adilia Warris

**Affiliations:** 1 MRC Centre for Medical Mycology, University of Exeter, Exeter EX4 4QD, UK; e.chesshyre@exeter.ac.uk; 2 Department of Paediatrics, Royal Devon University Healthcare NHS Foundation Trust, Exeter EX2 5DW, UK; 3 Faculty of Health and Life Sciences, University of Exeter, Exeter EX4 4QJ, UK; f.c.warren@exeter.ac.uk (F.C.W.); a.c.shore@exeter.ac.uk (A.C.S.); 4 NIHR Exeter Clinical Research Facility, Royal Devon University Healthcare NHS Foundation Trust, Exeter EX2 5DW, UK; 5 National Heart and Lung Institute, Imperial College, London SW3 6LY, UK; j.c.davies@imperial.ac.uk; 6 Royal Brompton and Harefield Hospitals, London SW3 6NP, UK; d.armstrong@imperial.ac.uk; 7 Department of Infectious Diseases, Imperial College, London W12 0BZ, UK; 8 Department of Paediatric Infectious Diseases, Great Ormond Street Hospital, London WC1N 3JH, UK

**Keywords:** *Aspergillus*, ABPA, cystic fibrosis, paediatrics, long-term outcomes, lung function

## Abstract

Observational studies indicate that *Aspergillus* colonization and allergic bronchopulmonary aspergillosis (ABPA) in people with cystic fibrosis (CF) are associated with poorer lung health and increased disease severity. We performed a longitudinal observational cohort study to analyse long-term outcomes of *Aspergillus* colonization and ABPA in children with CF. Anonymised UK CF Registry data from 2009 to 2019 for patients aged 8–17 years in 2009–2010 were collected. For the baseline cohort analysis, patients were classified based on the presence of *Aspergillus* colonization and ABPA in 2009 and/or 2010. For the longitudinal analysis, patients were categorised according to annual *Aspergillus* colonization and ABPA status. Comparisons made were (1) *Aspergillus* positive vs. negative; (2) excluding those with ABPA: *Aspergillus* positive vs. negative; and (3) ABPA positive vs. negative. Primary outcome was percentage predicted FEV_1_ decline and secondary outcomes included BMI decline, mortality, lung transplant, and IV antibiotic use. Of the 1675 children, 263 had *Aspergillus* colonization in the baseline cohort, 260 were diagnosed with ABPA, and 80 had both. Baseline cohort analysis showed significantly lower lung function (*p* < 0.0001) and increased antibiotic treatment (*p* < 0.001) in those with *Aspergillus* colonization and in those with ABPA. Longitudinal analysis showed ABPA was associated with increased decline in lung function (*p* < 0.00001) and BMI (*p* < 0.00001). *Aspergillus* colonization was associated with increased decline in BMI (*p* = 0.005) but not lung function (*p* = 0.30). ABPA was associated with increased decline in long-term lung function and BMI in children and young people with CF. *Aspergillus* colonization was associated with lower lung function at baseline, but no increased rate of decline was observed long-term.

## 1. Introduction

Cystic fibrosis (CF) is characterised by recurrent respiratory infections causing long-term morbidity and mortality. The importance of diagnosis and management of bacterial infections to preserve lung function is well appreciated and international guidance reflects this [1,2]. Fungal respiratory infections, of which *Aspergillus* is the commonest, have received less attention and their role in CF lung disease progression is less understood. *Aspergillus*, of which *Aspergillus fumigatus* is the most frequently encountered species, causes both non-allergic and allergic disease in CF. Allergic bronchopulmonary aspergillosis (ABPA) can lead to fibrotic lung disease and bronchiectasis [3]. The significance of *Aspergillus* colonization in CF is less clear and clinical management guidance is lacking [4,5]. Furthermore, the impact of highly effective CFTR modulator therapy, which dramatically improves lung function and quality of life in CF [6,7], on *Aspergillus* colonization and ABPA is currently unknown.

In 2021, UK clinicians reported ABPA in 3.1% of under 16-year-olds [8]; whereas a worldwide meta-analysis reported a rate of 8.9% in children with CF [9]. Comparison of prevalence of ABPA across studies is difficult due to the multiple clinical diagnostic criteria used; the lack of a single easily available diagnostic marker; and variable practices used regionally in screening for ABPA, despite two international consensus guidelines for the diagnosis and treatment of ABPA being available [10,11]. Whilst long-term complications of ABPA have been well described, large CF registry studies have so far not shown an association of ABPA with increased lung function decline over time [12,13]. A small study showed an association of increased lung function decline with ABPA (*n* = 34), with *Aspergillus* sensitisation (*n* = 63), and in those with persistent positive *Aspergillus* culture (*n* = 37), after adjustment for confounders [14]. Kraemer et al. showed an association of ABPA in children with CF (*n* = 16) and progression of a variety of lung function parameters indicative of airway narrowing, gas trapping, and small airway disease [15].

Reported rates of *Aspergillus*-positive respiratory samples in CF patients vary from 10% to 57%, reflecting differences in patient characteristics (age, disease severity), respiratory sampling (type and frequency), culture methods, and treatment regimens [16,17]. *Aspergillus* colonization (≥1 positive respiratory culture for *Aspergillus*/year) increases throughout childhood, with UK CF Registry (UKCFR) data showing that in 12- to 15-year-olds, 9.6% were colonized with *Aspergillus* [8]. To what extent *Aspergillus* colonization causes clinical symptoms and/or lung function decline is not fully understood. Small longitudinal observational studies comparing lung function decline in CF adults and children with and without persistent *Aspergillus* colonization have shown mixed results with an association shown in some studies (*n* = 251; *n* = 230) [14,18], but not in other studies (*n* = 121; *n* = 437) [19,20]. A challenge in comparing studies is the difference in definitions used for *Aspergillus* colonization which varies between ≥1 positive respiratory sample/year [21]; ≥2 positive respiratory samples/year [18,19,22]; or ≥3 positive cultures in 6 months, each separated by 1 month [14]. According to the Leeds criteria originally devised for *Pseudomonas aeruginosa* chronic infection, a differentiation is made between categories 0–3, which needs a minimum number of four respiratory samples cultured/year: 0: never cultured; 1: not grown in previous 12 months having previously been positive; 2: intermittent with ≤50% of samples positive in 12 months; and 3: chronic infection with >50% positive samples in 12 months [5,23,24]. The term *Aspergillus* colonization is usually used as it is difficult to differentiate colonization from infection (e.g., causing respiratory symptoms) due to the omnipresence of *Aspergillus* in the air [14,18]. Larger cross-sectional national CF Registry studies have also shown variable results with two showing an association between *Aspergillus* colonization and lower lung function (*n* = 9270; *n* = 770) [21,24], and another showing no association between persistent *Aspergillus* colonization and worse outcomes (*n* = 749) [5]. A recent, small longitudinal study (*n* = 119) showed an association between the presence of *Aspergillus* in BAL samples in young children with CF and greater lung function decline [25], while an earlier similar study (*n* = 156) did not show such an association [26]. A recent observational study in children aged 0 to 6 years (*n* = 330) showed an association with *Aspergillus*-positive BAL samples and progression of structural lung disease and increased risk of respiratory admission [27].

The conflicting results of the relatively small longitudinal and larger cross-sectional studies on long-term outcomes of *Aspergillus* colonization, and the lack of robust data demonstrating an association of lung function decline and ABPA, necessitates a large longitudinal study. We therefore aimed to assess the impact of *Aspergillus* colonization and/or ABPA on long-term outcomes in children with CF.

## 2. Methods

### 2.1. Study Population and Design

Anonymised longitudinal annual review data from 2009 to 2019 were provided by the UKCFR for patients with CF aged between 8 and 17 years in 2009 and 2010 (see Supplementary Materials for the full list of variables). The UKCFR is a research ethics committee-approved research database which holds data in a secure centralized database from people with CF in the UK on clinical and demographic characteristics upon informed consent. Under-8-year-olds were excluded due to the challenges in reliable lung function testing and obtaining sputum samples. The years 2009 and 2010 were chosen as the baseline years as complete data were recorded in the UKCFR for the first time for over 80% of patients with CF in 2009. Patients were excluded if no respiratory samples were taken in either 2009 or 2010, or if they did not survive after 31 December 2010. The study was approved by the UKCFR Steering Committee (Huntingdon Research Ethics Committee [07/Q0104/2]). For the baseline cohort analysis, patients were classified based on the presence of *Aspergillus* colonization (defined as ≥1 positive respiratory culture/year) or ABPA in 2009 and/or 2010. For the longitudinal analysis, patients were then categorized annually, according to their *Aspergillus* colonization status (defined as ≥1 positive respiratory culture in the preceding year) and ABPA in the preceding year up until annual review. The following comparisons were made: (1) *Aspergillus* positive vs. negative; (2) excluding those with ABPA: *Aspergillus* positive vs. negative; and (3) ABPA positive vs. negative. An additional longitudinal analysis was completed according to the presence or absence in one of the baseline years (2009–2010) of *Aspergillus* colonization (≥1 positive respiratory culture in 2009 and/or 2010) or ABPA (ABPA in 2009 and/or 2010).

The definition of ABPA used by the UKCFR is that of the CF Foundation Consensus Criteria [10]. *Aspergillus* colonization was defined as ≥1 positive respiratory sample since last annual review as per the UKCFR reporting on *Aspergillus* [8]. We were not able to use the Leeds criteria [23] or distinguish between isolated and persistent *Aspergillus* colonization in 12 months as the UKCFR did not capture the total number of respiratory samples and number of positive cultures per year at the time of the study. Information about the type of respiratory samples, frequency of respiratory sampling per year, number of positive samples per year, *Aspergillus* species, and use of antifungal treatment was not recorded in the UKCFR. The use of intravenous (IV) antibiotics was used as a proxy for frequency of pulmonary exacerbations.

The primary outcome was decline in lung function measured as percentage predicted FEV_1_ (ppFEV_1_) (Global Lung Initiative Network) [8]. Secondary outcomes included decline in percentile BMI (pBMI); IV antibiotic use; mortality; and lung transplantation. Sub-analyses were performed for age at baseline (childhood (8–11 years), adolescence (12–17 years)).

### 2.2. Statistical Analysis

#### 2.2.1. Baseline Characteristics: Cross-Sectional Analysis

Descriptive analysis was performed for the baseline cohort analysis using the Chi squared test (categorical variables), independent *t*-test, or Mann–Whitney U test (continuous variables). Linear (continuous outcome), logistic (binary outcome), and negative binomial (count outcomes) regression models were then used to assess whether differences at baseline were independent of the known confounders of age, sex, *CFTR* genotype (F508del homozygous, F508del heterozygous and other), and *Pseudomonas* co-infection. These were chosen as all have a well-described impact on long-term outcomes in CF [28]. Results are presented with 95% confidence intervals (CI). A significance threshold of 0.05 was used. No formal adjustments for multiple testing were made. The analyses of the primary outcome (ppFEV_1_) were interpreted first, with additional analyses interpreted in the light of multiple testing.

#### 2.2.2. Longitudinal Analysis

Longitudinal outcomes were analysed according to *Aspergillus* colonization/ABPA status as time-varying throughout the cohort, i.e., according to the *Aspergillus* colonization/ABPA status in the year preceding annual review. For the *Aspergillus* colonization/ABPA time-varying analyses for lung function and BMI outcomes, a linear mixed-effects regression model was used, with mean differences between groups presented. For intravenous (IV) antibiotic days, multi-level mixed-effects negative binomial regression models were used, with odds ratios (ORs) presented. For death and lung transplant outcomes, complementary log–log regression models were used, with ORs presented. A sensitivity analysis was performed on ‘completed cases’ only, to assess the impact of missing data.

An additional longitudinal analysis was performed according to baseline *Aspergillus* colonization/ABPA status (i.e., presence or absence of *Aspergillus* colonization and ABPA in 2009 and/or 2010) for lung function and BMI outcomes, for which linear mixed-effect regression models were used (lung transplant excluded); and for death and lung transplant outcomes Cox’s proportional hazards models with hazard ratios (HRs) are presented.

All results are presented with 95% CI. Univariable and multivariable analysis were completed for all models, with adjustment in the multivariable models for the known confounders including age, sex, *CFTR* genotype at baseline; and *Pseudomonas* infection, lung function (if not the outcome), and BMI (if not the outcome), and ABPA (where ABPA was not included as a predictor) in the same year. For the longitudinal models according to the baseline *Aspergillus* colonization/ABPA groups, additional adjustments for nontuberculous mycobacteria and CF-related diabetes (CFRD) treatment at baseline were made. Lung transplant patients were excluded from the linear mixed-effect regression models. Due to a high amount of missing data for the CFRD treatment variable, and for the purposes of this model, if the CFRD treatment data field was left blank, it was assumed that the patient was not on CFRD treatment.

Sub-group analyses were performed using the models outlined above on patients aged 8 to 11 years (childhood group) and those aged 12 to 17 years (adolescent group) at baseline to evaluate the impact of *Aspergillus* colonization and ABPA in childhood and adolescence separately.

Statistical analysis was performed using Stata version 18. Statistical significance was assumed at *p* < 0.05.

## 3. Results

### 3.1. Baseline Characteristics

A total of 1675 patients (ethnicity 96% white) were assessed according to baseline *Aspergillus* colonization in 2009 and/or 2010 and ABPA status in 2009 and/or 2010 (subjects could be in more than one analysis): (1): *Aspergillus* (*n* = 263) vs. no-*Aspergillus* (*n* = 1412); (2) ABPA excluded: *Aspergillus* (*n* = 183) vs. no-*Aspergillus* (*n* = 1232); (3) ABPA (*n* = 260) vs. no-ABPA (*n* = 1415) (Table 1).

Baseline characteristics are presented in Table 2 and Table 3. There were no significant differences at baseline in terms of age, gender, or *CFTR* genotype. Lung function (mean ppFEV_1_) was significantly lower in the *Aspergillus* and ABPA groups compared to the respective control groups (Table 2: *Aspergillus* vs. no-*Aspergillus*: 71.1% vs. 78.9%; *p* < 0.00001; excluding ABPA: *Aspergillus* vs. no-*Aspergillus*: 71.4% vs. 79.7%; *p* < 0.00001; and ABPA vs. no-ABPA: 72.5% vs. 78.6%; *p* < 0.00001). These results were confirmed in the multivariable analysis adjusted for the key confounders age, gender, *CFTR* genotype, and *P. aeruginosa* co-infection (Table 3).

Mean pBMI was significantly lower in the *Aspergillus* colonization groups compared to their control groups, but not in the ABPA vs. no-ABPA group (Table 2): *Aspergillus* vs. no-*Aspergillus*: 45.7 ± 28.2 vs. 50 ± 29.6 (*p* = 0.03); excluding ABPA: *Aspergillus* vs. no-*Aspergillus*: 44.3 ± 28.3 vs. 49.7 ± 29.8 (*p* = 0.02). These results were confirmed in the multivariable analysis adjusted for confounders (Table 3).

Multivariable analyses showed increased use of IV antibiotics in both the *Aspergillus* colonization and ABPA groups compared to the respective control groups (*p* < 0.0001) with length of IV antibiotic course significantly increased in the *Aspergillus* groups with or without ABPA (*p* = 0.007 and *p* = 0.003, respectively). Oral steroid use was significantly increased in the ABPA vs. no-ABPA group (*p* = 0.001). Those with *Aspergillus* colonization and ABPA had higher rates of chronic macrolides and nebulised anti-pseudomonal antibiotics use than those without (Table 3).

Increased rates of *P. aeruginosa*, *Stenotrophomonas* spp., and non-tuberculous mycobacteria were observed in all the *Aspergillus* colonization and ABPA baseline groups compared to the respective control groups (Table 3). There were significantly increased rates of *Staphylococcus aureus* in those with *Aspergillus* compared to those without, but not in those with ABPA compared to those without (Table 3).

There were no significant differences between the *Aspergillus* colonization and ABPA groups compared to the respective control groups in rates of non-invasive ventilation or lung transplant (Table 2). No patients at baseline were on CFTR modulator treatment.

### 3.2. Long-Term Outcomes

By the end of 2019, 101 (6.0%) patients had died, 62 (3.7%) had received a lung transplant, and 149 (8.9%) were no longer recorded in the UKCFR. In 2013, the first 55 patients were on CFTR modulator treatment, which had increased to 196 patients by 2019. The use of CFTR modulator treatment did not differ according to *Aspergillus* colonization and ABPA status at annual review (Supplemental Table S5).

**Figure 1:**
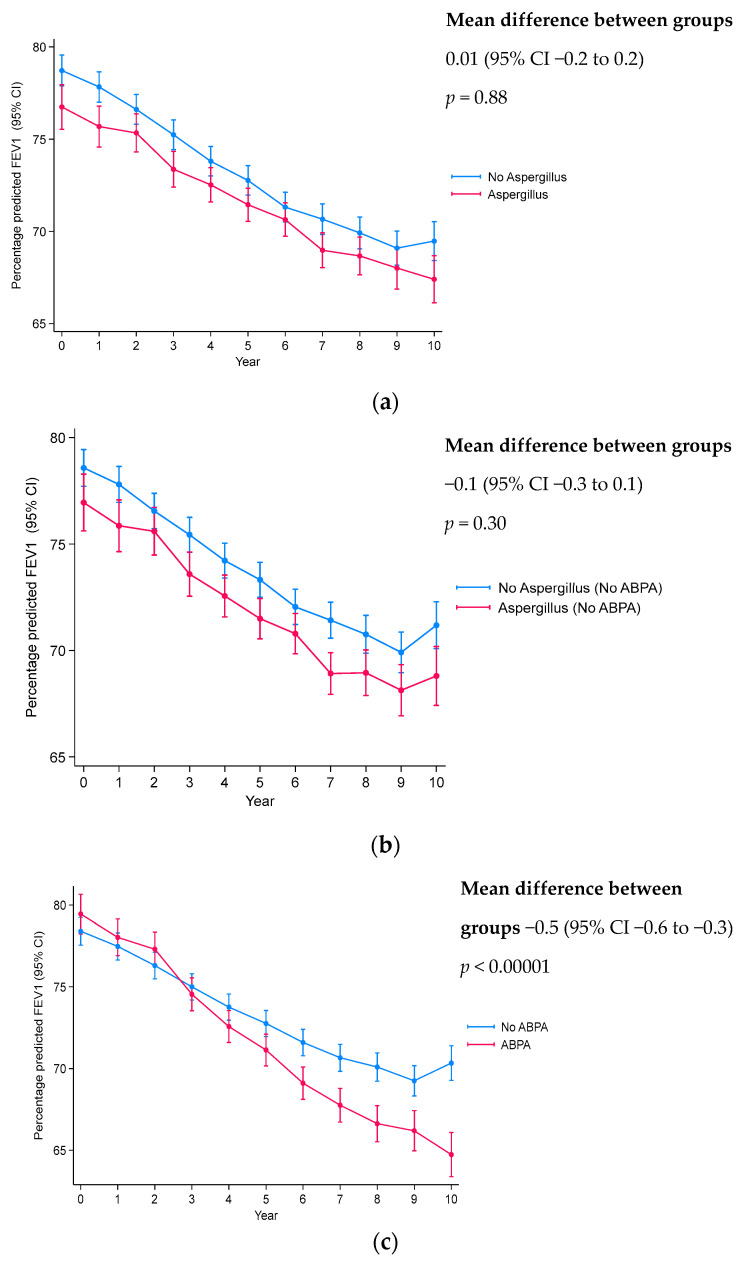
Lung function (ppFEV1) decline according to *Aspergillus* colonization and ABPA status each year. (**a**) *Aspergillus* colonization vs. no-*Aspergillus*. (**b**) Excluding ABPA: *Aspergillus* colonization vs. no-*Aspergillus*. (**c**) ABPA vs. no-ABPA. Linear mixed-effects multivariable analysis with adjustment for confounders: age, sex, *CFTR* genotype, *Pseudomonas aeruginosa* co-infection, percentile BMI, and ABPA (Figure 1a only). *P* value indicates significance between slopes of decline.

**Figure 2:**
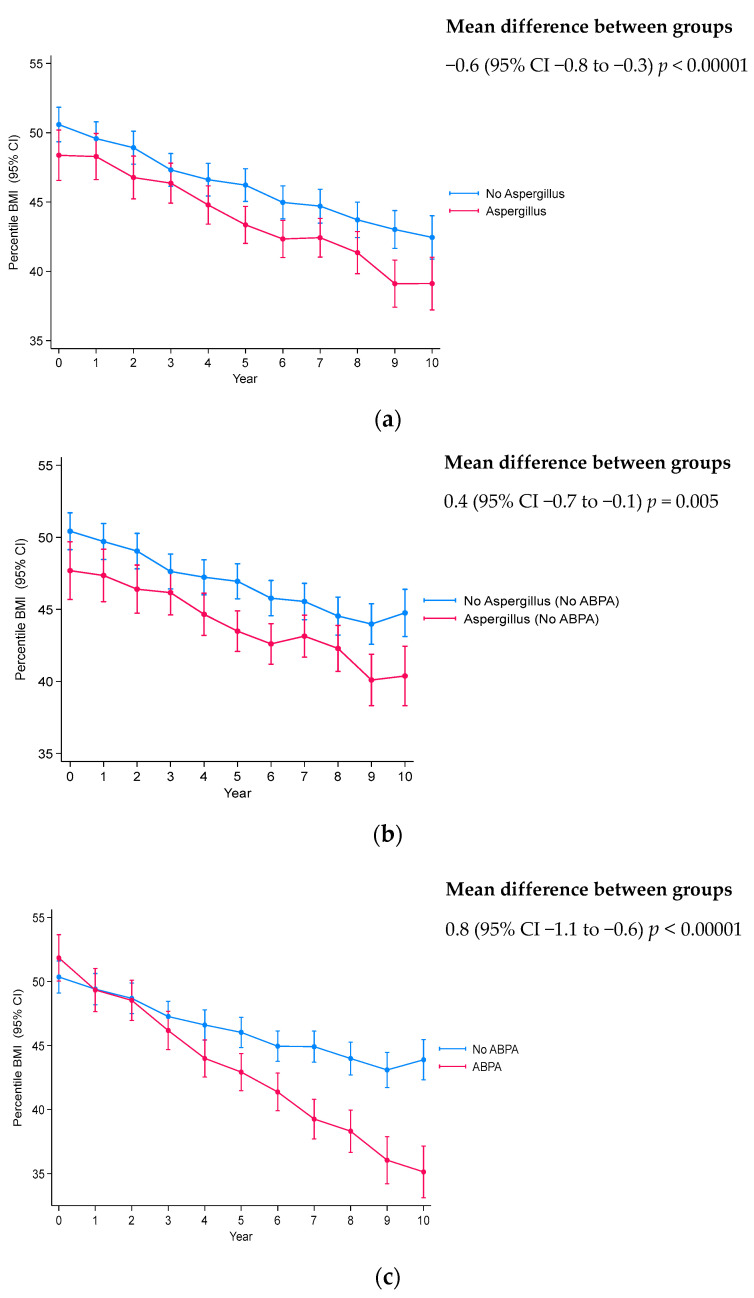
Percentile body mass index (BMI) decline according to *Aspergillus* colonization and ABPA status each year. (**a**) *Aspergillus* colonization vs. no-*Aspergillus*. (**b**) Excluding ABPA: *Aspergillus* colonization vs. no-*Aspergillus*. (**c**) ABPA vs. no-ABPA. Linear mixed-effects multivariable analysis with adjustment for confounders: age, sex, *CFTR* genotype, *Pseudomonas aeruginosa* co-infection, percentage predicted FEV_1_, and ABPA (Figure 2a only). *p*-value indicates significance between slopes of decline.

Longitudinal analysis showed a significantly lower lung function in those with *Aspergillus* colonization and ABPA compared to those without (Figure 1; Table 4). Although, a significantly increased lung function decline was only observed in those with ABPA vs. those without (mean difference between groups −0.5 (−0.6 to −0.3); *p* < 0.00001). Sub-analyses according to age at baseline show a significant increase in lung function decline in both children and adolescents with ABPA compared to no-ABPA (*p* < 0.00001 and *p* = 0.01, respectively, Supplemental Tables S2 and S3), with no significant differences in the *Aspergillus* vs. no-*Aspergillus* colonization groups in either the childhood or adolescent sub-groups. pBMI decline was significantly increased in those with *Aspergillus* colonization, regardless of ABPA exclusion (mean difference between groups −0.6 (−0.8 to −0.3), *p* < 0.00001 and mean difference between groups −0.4 (−0.7 to −0.1), *p* = 0.005, respectively), and those with ABPA (mean difference between groups −0.8 (−1.1 to −0.6), *p* < 0.00001) (Figure 2, Table 4). Sub-group analysis showed increased rate of pBMI decline in all those with *Aspergillus* colonization and/or ABPA, except for adolescents with *Aspergillus* colonization and ABPA excluded (Supplemental Tables S2 and S3).

There was no increase in the frequency of IV antibiotic days, and there were no differences in mortality or number of lung transplants, over the follow-up in either the *Aspergillus* colonization or ABPA groups (Table 4, Tables S1–S3). The sensitivity analysis performed with complete cases only did not impact the outcomes (Table S4).

Longitudinal analysis according to *Aspergillus* colonization/ABPA status at baseline shows similar results to the *Aspergillus* colonization/ABPA time-varying analyses with a significant decline in lung function in those with ABPA at baseline (*p* = 0.003) (Table S1). BMI decline was also significantly increased in the ABPA group (*p* < 0.0001).

## 4. Discussion

This is the largest and longest cohort study to investigate the long-term outcomes of *Aspergillus* colonization and ABPA in children and young people with CF. Our data show that ABPA was significantly associated with a faster decline in lung function and BMI, whereas *Aspergillus* colonization is only associated with increased decline in BMI.

It is generally accepted that ABPA causes lung damage in people with CF and treatment strategies are well established [10,11]. However, no previous large, longitudinal studies have demonstrated a detrimental effect on lung function over time. A European CF Society Patient Registry showed that young people with CF and ABPA (3550 aged 6–17 years) had significantly lower lung function compared to those without ABPA at baseline and at the 2-year follow-up, with no increased decline in the ABPA group compared to the non-ABPA group [12]. The 5-year longitudinal study from the European Epidemiologic Registry of CF with 12,447 paediatric and adult patients also showed no increased lung function decline over time in those with and without ABPA [13]. The shorter follow-up periods might be a reason for the lack of observed increase in lung function decline in these large studies. Our findings, showing increased long-term decline in lung function and BMI in children and young people with ABPA after adjustment for confounders, provide robust longitudinal evidence of the long-term effects of ABPA and demonstrate the importance of early diagnosis and treatment. The fact that the results of our longitudinal analyses, according to the presence of ABPA at baseline, show a significantly increased decline in both lung function and BMI in those with ABPA compared to those without, underscores the need for early intervention.

The significant association of *Aspergillus* colonization and increased BMI decline (a key determinant of outcome in CF), demonstrates the importance of *Aspergillus* colonization. The lack of association with increased lung function decline may have been partly due to the definition of *Aspergillus* colonization used in this study (≥1 positive sample/year), and lack of data collected by the UKCFR at the time on respiratory sample type and number of samples. Consequently, the *Aspergillus* colonization groups in our study include both patients with a single positive *Aspergillus* culture per year, as well as those with multiple positive cultures. The diagnostic sensitivity of a positive culture varies hugely depending on the respiratory sample type and culture methods used. Sputum samples and BAL fluids have a higher diagnostic yield for *Aspergillus* compared to cough and throat swabs [29,30]. In addition, standard culture-based methods can fail to detect *Aspergillus,* and high-volume cultures have shown to increase the yield of *Aspergillus* detection in respiratory samples [31]. There is an urgent need to standardize the definition of *Aspergillus* colonization and infection, with standardization of diagnostic methods (microbiology cultures, biomarkers, imaging, etc.), to be able to identify diseases associated with *Aspergillus* colonization, to identify characteristics of people who may benefit from antifungal treatment, and to allow comparison of outcomes between studies [29,30]. A study of 132 CF patients showed greater lung function decline in those positive for *Aspergillus* over consecutive years compared to single year positive for *Aspergillus* [32].

In our study, the impact of *Aspergillus* colonization on BMI decline was most pronounced in childhood as shown by our sub-analysis, suggesting that the impact of *Aspergillus* colonization is more severe at younger ages. The significance of early, positive BAL-fluid cultures for *Aspergillus* in infancy has previously been shown in the ACF-BAL cohort of 119 infants with CF and was associated with reduced lung function at the age of 12 years [25]. Another study on 53 infants from the AREST-CF cohort showed that in the 13% with positive *Aspergillus* BAL-fluid cultures, there was a 11.3% reduction in FEV_0.75_ (−18.9 to −3.1; *p* < 0.01) in children aged 4 to 8 years [33]. Other studies from the AREST-CF cohort (*n* = 330) underpin the association between early, positive BAL-fluid cultures for *Aspergillus* in childhood CF and the progression of structural lung disease [26,27].

Unfortunately, we were unable to assess the impact of antifungal treatment as antifungal use was not captured at the time by the UKCFR. National and international surveys have shown that clinical practice is highly variable with respect to antifungal prescriptions for CF-related *Aspergillus* colonization and disease [4,34]. It remains to be shown if early treatment of *Aspergillus* colonization with antifungals and upfront treatment of ABPA with antifungals in addition to corticosteroids can improve outcomes. In addition, the impact of highly effective CFTR modulator therapies on *Aspergillus* lung disease in CF is as yet unclear.

Our cross-sectional analysis of the baseline cohort shows that both the *Aspergillus* colonization and ABPA groups had increased co-infections (*P. aeruginosa*, *Stenotrophomonas* spp., and nontuberculous mycobacteria) compared with the no-*Aspergillus* and no-ABPA groups. The high rate of *P. aeruginosa* co-infection has been shown in other cross-sectional CF registry studies [5]. Recently in a cross-sectional UKCFR study, Hughes et al. [21] showed a rate of 9.1% (846/9270) of *P. aeruginosa–Aspergillus* co-infection. In the study by Hughes et al., while *P. aeruginosa–Aspergillus* co-infection was not associated with reduced lung function compared to *P. aeruginosa* alone, it was associated with increased use of antibiotics [21]. The strong association shown in our study of *Aspergillus* colonization with IV antibiotics at baseline either likely reflected underlying *P. aeruginosa* infection and other co-infections, and consequently worse lung disease pre-disposing the patients to *Aspergillus* colonization; or may reflect antibiotic treatment pre-disposing them to *Aspergillus* colonization. The strong association of *Aspergillus* and *P. aeruginosa* demonstrates the importance of adjustment for *P. aeruginosa* infection which was performed for all our multivariable analyses. Our longitudinal analysis on IV antibiotic use shows that the high rate of IV antibiotic days in those with *Aspergillus* colonization/ABPA at baseline does not increase over time but remains high compared to those without *Aspergillus*/ABPA. As IV antibiotic days are a surrogate marker for pulmonary exacerbation rate [18,30], this indicates that *Aspergillus* colonization and ABPA are associated with increased pulmonary exacerbations, but the difference does not increase over time.

A key challenge in any registry study is missing data. Other than data about the CFRD treatment, missing data were <15% in all but the last year of the study. To assess the potential impact of missing data on the outcomes, a sensitivity analysis was performed on ‘complete cases’ only for the time-varying *Aspergillus* colonization/ABPA longitudinal models and did not show any difference in results. Data on CFRD treatment were missing in >60% at baseline. Therefore, as part of the longitudinal analysis according to baseline *Aspergillus* colonization/ABPA status, we adjusted for CFRD treatment at baseline by assuming that any missing data indicated that the patient was not on CFRD treatment (as opposed to where a yes or no was recorded). This adjustment did not change the overall results of this analysis.

In terms of outcomes, while ppFEV_1_ is the most widely used measure of lung function decline, other lung function parameters such as FEF_50_ (marker of small airway disease), sReff (marker of airway narrowing), V_TG_ (marker of trapped gas), and FRCpleth (marker of pulmonary hyperinflation), as well as lung clearance index (LCI) (measure of ventilation homogeneities) are of value to give a more detailed insight into the extent of airway disease [15]. However, such detailed lung function data are rarely available from Registry data, as in our study.

In conclusion: this is the first large longitudinal study to demonstrate increased lung function and BMI decline in children and adolescents with CF and ABPA. The association of *Aspergillus* colonization with increased BMI decline is of interest and indicates that non-allergic *Aspergillus* colonization is of clinical importance.

## Figures and Tables

**Table 1:** *Aspergillus* colonization and/or ABPA at baseline (2009/2010) in the study cohort.

	*Aspergillus* Positive	*Aspergillus* Negative	Total
ABPA present	*n* = 80	*n* = 180	*n* = 260
ABPA absent	*n* = 183	*n* = 1232	*n* = 1415
Total	*n* = 263	*n* = 1412	*n* = 1675

**Table 2:** Comparison of baseline characteristics of the study population in 2009/2010 according to *Aspergillus* colonization and ABPA status.

		*Aspergillus* Colonization versus No-*Aspergillus*			ABPA Excluded: *Aspergillus* Colonization versus No-*Aspergillus*			ABPA versus No-ABPA		
		No-*Aspergillus* (*n* = 1412)	*Aspergillus* (*n* = 263)	*p*-Value	ABPA Excluded: No-*Aspergillus* (*n* = 1232)	ABPA Excluded: *Aspergillus* (*n* = 183)	*p*-Value	No-ABPA (*n* = 1415)	ABPA (*n* = 260)	*p*-Value
Male sex, n/total (%)		717/1412 (50.8%)	134/263 (51.0%)	*p* = 0.96	615/1232 (49.9%)	94/183 (51.4%)	*p* = 0.72	709/1415 (50.1%)	142/260 (54.6%)	*p* = 0.18
Age in years (2009), mean (SD; range)		12.0 (2.5; 8–17)	12.1 (2.5; 8–17)	*p* = 0.40	11.9 (2.5, 8–17)	12.0 (2.5, 8–16)	*p* = 0.61	11.9 (2.5, 8–17)	12.3 (2.5, 8–17)	*p* = 0.05
*CFTR* genotype, n/total (%)				*p* = 0.99			*p* = 0.64			*p* = 0.82
	F508del homozygous	783/1399 (56.0%)	147/262 (56.1%)		677/1220 (55.5%)	106/182 (58.2%)		783/1402 (55.9%)	147/259 (56.8%)	
	F508del heterozygous	518/1399 (37.0%)	96/262 (36.6%)		456/1220 (37.4%)	66/182 (36.3%)		522/1402 (37.2%)	92/259 (35.5%)	
	Other	98/1399 (7.0%)	19/262 (7.3%)		87/1220 (7.1%)	10/182 (5.5%)		97/1402 (6.9%)	20/259 (7.7%)	
ppFEV_1_ (2010), mean (SD; range)		78.9 (18.3, 14.9–125.9) (*n* = 1280)	71.1 (18.7, 20.9–118.4) (*n* = 243)	*p* < 0.00001	79.7 (18.2, 14.9–125.9) (*n* = 1108)	71.4 (19.3, 20.9–118.4) (*n* = 170)	*p* < 0.00001	78.6 (18.5, 14.9–125.9) (*n* = 1278)	72.5 (18.1, 15.9–116.7) (*n* = 245)	*p* < 0.00001
pBMI (2010), mean (SD; range)		50.0 (29.6, 0–99.9) (*n* = 1372)	45.7 (28.2, 0–99.5) (*n* = 258)	*p* = 0.03	49.7 (29.8, 0–99.9) (*n* = 1195)	44.3 (28.3, 0–99.5) (*n* = 180)	*p* = 0.02	49.0 (29.7, 0–99.9) (*n* = 1375)	51.1 (27.9, 0.1–99.2) (*n* = 255)	*p* = 0.30
**Respiratory disease severity markers**										
Oxygen therapy in 2010, n/total (%)		57/1342 (4.3%)	15/246 (6.1%)	*p* = 0.20	43/1168 (3.7%)	13/171 (7.6%)	*p* = 0.02	56/1339 (4.2%)	16/249 (6.4%)	*p* = 0.12
Non-invasive ventilation in 2010, n/total (%)		21/1326 (1.6%)	2/244 (0.8%)	*p* = 0.36	16/1154 (1.4%)	1/169 (0.6%)	*p* = 0.39	17/1323 (1.3%)	6/247 (2.4%)	*p* = 0.17
Evaluation for lung transplant, n/total (%)		14/1359 (1.0%)	3/250 (1.2%)	*p* = 0.81	12/1185 (1.0%)	3/172 (1.7%)	*p* = 0.39	15/1357 (1.1%)	2/252 (0.8%)	*p* = 0.66
Lung transplant, n/total (%)		10/1412 (0.7%)	0/263 (0.0%)	*p* = 0.17	10/1232 (0.8%)	0/183 (0%)	*p* = 0.22	10/1415 (0.7%)	0/260 (0%)	*p* = 0.17

ppFEV_1_ = percentage predicted forced expiratory volume in 1 s. pBMI = percentile body mass index. Categorical variables: numbers (%). *p*-value calculated with Chi-squared test. Continuous variables: mean (SD, range). *p*-value calculated with independent sample *t*-test unless otherwise stated. Mann–Whitney U test. No missing data, except where specified.

**Table 3:** Cross-sectional analysis according to baseline (2009/2010) *Aspergillus*/ABPA status (Aspergillus colonization in 2009 and/or 2020 and ABPA in 2009 and/or 2010). Multivariable analysis presented, with adjustments for key confounders *.

	*Aspergillus* Colonization versus No-*Aspergillus*	ABPA Excluded: *Aspergillus* Colonization versus No-*Aspergillus*	ABPA versus No-ABPA
**Clinical characteristics (2010)**			
ppFEV_1_, mean difference (95% CI)	−6.3 (−8.8 to −3.9) *p* < 0.0001 (*n* = 1513) **	−6.9 (−9.8 to −4.1) *p* < 0.0001 (*n* = 1269)	−5.0 (−7.4 to −2.5) *p* < 0.0001 (*n* = 1513)
pBMI, mean difference (95% CI)	−3.5 (−7.5 to 0.5) *p* = 0.08 (*n* = 1617)	−4.5 (−9.2 to 0.2) *p* = 0.06 (*n* = 1363)	2.7 (−1.3 to 6.7) *p* = 0.18 (*n* = 1617)
**Bacterial infection (2009–2010)**			
*Pseudomonas aeruginosa,* OR (95% CI)	2.1 (1.6 to 2.7) *p* < 0.0001 (*n* = 1661)	1.7 (1.3 to 2.4) *p* = 0.001 (*n* = 1402)	1.6 (1.2 to 2.1) *p* = 0.001 (*n* = 1661)
Chronic *Pseudomonas aeruginosa,* OR (95% CI)	1.3 (0.9 to 1.7) *p* = 0.12 (*n* = 1490)	1.1 (0.8 to 1.6) *p* = 0.55 (*n* = 1251)	1.4 (1.1 to 1.9) *p* = 0.02 (*n* = 1490)
*Burkholderia* spp., OR (95% CI)	1.2 (0.6 to 2.5) *p* = 0.63 (*n* = 1661)	1.1 (0.4 to 2.5) *p* = 0.91 (*n* = 1402)	0.6 (0.2 to 1.4) *p* = 0.23 (*n* = 1661)
*Stenotrophomonas* spp., OR (95% CI)	3.3 (2.0 to 5.3) *p* < 0.0001 (*n* = 1661)	4.4 (2.5 to 7.8) *p* < 0.0001 (*n* = 1402)	2.4 (1.5 to 4.0) *p* = 0.001 (*n* = 1661)
*Staphylococcus aureus,* OR (95% CI)	1.5 (1.2 to 2.0) *p* = 0.004 (*n* = 1661)	1.6 (1.2 to 2.2) *p* = 0.005 (*n* = 1402)	0.9 (0.7 to 1.2) *p* = 0.56 (*n* = 1661)
*Haemophilus influenza B,* OR (95% CI)	1.5 (0.9 to 2.3) *p* = 0.09 (*n* = 1661)	1.4 (0.8 to 2.4) *p* = 0.19 (*n* = 1402)	0.7 (0.4 to 1.2) *p* = 0.23 (*n* = 1661)
Non-tuberculous mycobacteria, OR (95% CI)	4.4 (2.4 to 8.1) *p* < 0.0001 (*n* = 1661)	8.0 (3.8 to 16.9) *p* < 0.0001 (*n* = 1402)	3.0 (1.6 to 5.6) *p* < 0.0001 (*n* = 1661)
**Steroid use (2009–2010)**			
Oral steroids, OR (95% CI)	1.8 (1.3 to 2.6) *p* = 0.001 (*n* = 1661)	1.0 (0.6 to 1.8) *p* = 0.89 (*n* = 1402)	7.7 (5.6 to 10.6) *p* < 0.0001 (*n* = 1661)
Inhaled steroids, OR (95% CI)	1.3 (1.0 to 1.7) *p* = 0.09 (*n* = 1661)	1.4 (1.0 to 2.0) p = 0.04 (*n* = 1402)	1.2 (0.9 to 1.6) *p* = 0.2 (*n* = 1661)
**Antibiotic use (2009–2010)**			
IV antibiotic requirement (yes/no), OR (95% CI)	3.7 (2.6 to 5.4) *p* < 0.0001 (*n* = 1525)	3.5 (2.4 to 5.3) *p* < 0.0001 (*n* = 1285)	4.4 (3.0 to 6.4) *p* < 0.0001 (*n* = 1525)
IV antibiotic days, IRR (95% CI)	1.2 (1.1 to 1.3) *p* = 0.007 (*n* = 920)	1.3 (1.1 to 1.5) *p* = 0.003 (*n* = 716)	1.1 (1.0 to 1.3) *p* = 0.09 (*n* = 920)
Oral antibiotics (chronic), OR (95% CI)	1.0 (0.8 to 1.4) *p* = 0.90 (*n* = 1661)	1.2 (0.8 to 1.6) *p* = 0.41 (*n* = 1402)	1.0 (0.8 to 1.3) *p* = 0.90 (*n* = 1661)
Oral macrolide (chronic), OR (95% CI)	1.6 (1.2 to 2.1) *p* = 0.001 (*n* = 1661)	1.5 (1.1 to 2.1) *p* = 0.02 (*n* = 1402)	2.2 (1.7 to 2.9) *p* < 0.0001 (*n* = 1661)
Oral flucloxacillin (chronic), OR (95% CI)	1.3 (1.0 to 1.6) *p* = 0.09 (*n* = 1661)	1.4 (1.0 to 1.9) *p* = 0.06 (*n* = 1402)	1.0 (0.8 to 1.4) *p* = 0.84 (*n* = 1661)
Nebulised tobramycin, OR (95% CI)	2.2 (1.6 to 3.0) *p* < 0.0001 (*n* = 1661)	2.1 (1.4 to 3.2) *p* < 0.0001 (*n* = 1402)	2.6 (1.9 to 3.6) *p* < 0.0001 (*n* = 1661)
Nebulised colistin/promixin, OR (95% CI)	1.9 (1.4 to 2.5) *p* < 0.0001 (*n* = 1661)	1.9 (1.3 to 2.7) *p* = 0.001 (*n* = 1402)	2.3 (1.7 to 3.2) *p* < 0.0001 (*n* = 1661)

ppFEV_1_ = percentage predicted forced expiratory volume in 1 s. pBMI = percentile body mass index. Chronic *P. aeruginosa* ≥ 2 isolates of *P. aeruginosa*/year. Multivariate linear and logistic regression and negative binomial models. Continuous variables: mean difference unless otherwise stated (95% CI), *p* value, (number). Categorical variables: Odds ratio (95% CI), *p* value, (number). Rates: IRR = incidence rate ratio (95% CI), *p* value, (number). * Adjusted for age, sex, *CFTR* genotype, *P. aeruginosa* co-infection at baseline. ** Numbers in brackets are the number of patients cited in analysis.

**Table 4:** Longitudinal analysis with time-varying predictors *Aspergillus* colonization and ABPA in the year preceding outcome at annual review: *Aspergillus* colonization (≥1 positive respiratory culture in preceding year) and ABPA in the preceding year. Mixed-effect regression models * showing multivariable analysis after adjustment for known confounders **.

Clinical Outcome	*Aspergillus* Colonization versus No-*Aspergillus* (*n* = 1675)	ABPA Excluded (Same Year): *Aspergillus* Colonization versus No-*Aspergillus* (*n* = 1675)	ABPA versus No-ABPA (*n* = 1675)
ppFEV_1_, between group mean difference (95% CI)	−0.01 (−0.2 to 0.2) *p* = 0.88 (*n* = 1650) ***	−0.1 (−0.3 to 0.1) *p* = 0.30 (*n* = 1611)	−0.5 (−0.6 to −0.3) *p* < 0.00001 (*n* = 1650)
pBMI, between group mean difference (95% CI)	−0.6 (−0.8 to −0.3) *p* < 0.00001 (*n* = 1650)	−0.4 (−0.7 to −0.1) *p* = 0.005 (*n* = 1611)	−0.8 (−1.1 to −0.6) *p* < 0.00001 (*n* = 1650)
IV antibiotic days (during 12 months prior to annual review), OR (95% CI)	1.3 (0.9 to 1.9) *p* = 0.86 (*n* = 1650)	1.3 (0.8 to 2.2) *p* = 0.93 (*n* = 1609)	2.2 (1.6 to 3.0) *p* = 0.67 (*n* = 1650)
Lung transplant (in 12 months prior to annual review), OR (95% CI)	0.7 (0.3 to 1.5) *p* = 0.34 (*n* = 1648)	0.7 (0.3 to 1.6) *p* = 0.43 (*n* = 1609)	0.2 (0.06 to 1.0) *p* = 0.05 (*n* = 1648)
Death (in 12 months prior to annual review), OR (95% CI)	0.9 (0.5 to 1.6) *p* = 0.59 (*n* = 1642)	0.8 (0.4 to 1.6) *p* = 0.49 (*n* = 1587)	1.4 (0.8 to 2.5) *p* = 0.26 (*n* = 1642)

ppFEV_1_ = percentage predicted forced expiratory volume in 1 s. pBMI = percentile body mass index. OR = Odds ratio, CI = confidence interval. * mixed-effect (random effect on patient) regression models (linear mixed-effect models (ppFEV1, pBMI); multilevel mixed-effect negative binomial models (IV antibiotic days); and complementary log–log regression model (death and lung transplant). ** Adjusted for known confounders: baseline age, sex, and *CFTR* genotype; and *P. aeruginosa* co-infection, ppFEV1 (where not outcome), pBMI (where not outcome), and ABPA (column 1 only) in the same year. *** Numbers in brackets are number of patients cited in the analyses.

## Data Availability

Dataset available on reasonable request.

## Supplementary Materials

The following supporting information can be downloaded at: https://www.mdpi.com/article/10.3390/jof10090599/s1, Table S1: Longitudinal analysis according to baseline (2009 and/or 2010) cohort classification of *Aspergillus* colonization and ABPA status; Table S2: Longitudinal childhood sub-group analysis (including only those aged 8 to 11 years old at baseline) with time varying *Aspergillus* and ABPA status at annual review; Table S3: Longitudinal adolescent sub-group analysis (including only those aged 12 to 17 years old at baseline) with time varying *Aspergillus* colonization and ABPA status at annual review; Table S4: Longitudinal sensitivity analysis on complete cases only with time varying *Aspergillus* colonization and ABPA status at annual review; Table S5: Proportion of patients on CFTR modulator therapy over the period of study (2009–2019) according to time-varying *Aspergillus* colonization and ABPA status at annual review.
