# Supplementary material for: Long-Term Outcomes of Allergic Bronchopulmonary Aspergillosis and Aspergillus Colonization in Children and Adolescents with Cystic Fibrosis
Source: J Fungi (Basel). 2024 Aug 24;10(9):599. doi: 10.3390/jof10090599 (PMC11433026; doi:10.3390/jof10090599)
Supplement: Supplementary file 1 [file jof-10-00599-s001.zip › jof-3093540-supplementary.pdf]

Supplemental file

Full list of variables as provided by the UK CF Registry

Fully anonymised data from the CF Registry was requested for the years 2009 to 2019 (01.01.09-31.12.19) from all participants aged 8 to 17 years in the years 2009-2010. Variables:

- Age at annual review encounter
- Diagnosis- age at diagnosis and presence of respiratory infection (acute or persistent)
- Gender
- Ethnicity
- CF gene mutation class
- BMI percentile (%)
- Baseline lung function as measured by $ppFEV_1$, and $ppFEV_1$ in subsequent years
- Total number of respiratory samples since last annual review (number in each category of sputum, cough/throat/nose, and bronchoscopy samples)
- *Aspergillus fumigatus* positive respiratory samples since last annual review Y/N and *Aspergillus* species positive respiratory samples since last annual review Y/N
- *Pseudomonas aeruginosa* (Pa) positive respiratory swabs that year Y/N
    - Mucoid/ non-mucoid
- Intermittent/ chronic Pa
- Other bacterial co-infections that year:
    - *Staphylococus aureus* Y/N (Chronic/intermittent)
    - *Burkholderia cepacia* complex Y/N
    - *Nontuberculous mycobacteria* Y/N
    - Other Y/N
- Number of hospitalisations for intravenous therapy (IV admissions)
- Number of intensive care unit admissions
- Number of home intravenous courses (home IV courses)
- Number of hospitalisations for non-intravenous therapy (non-IV admissions)
- Allergic bronchopulmonary aspergillosis (ABPA) Y/N
- Chronic treatment with antibiotics Y/N
    - Inhaled anti-pseudomonal antibiotics
    - Oral flucloxacillin
    - Oral anti-pseudomonal antibiotics
- Chronic treatment with steroids Y/N
- CFTR modulator therapy Y/N and which type: ivacaftor alone/lumacaftor- ivacaftor/ tezecaftor-ivacaftor/elexacaftor-tezacaftor-ivacaftor
- Oxygen therapy since last annual review: continuous, or nocturnal +/- with exertion, or during exacerbation or PRN

- Non-invasive ventilation therapy in the last year Y/N
- Presence of complications:
    - CF related diabetes (CFRD) Y/N
    - Pancreatic insufficiency Y/N
- Evaluation for lung transplantation in that year (accepted/declined/deferred) Y/N
- Lung transplant in that year Y/N
- Death Y/N
- Primary cause of death

Supplemental table S1: Longitudinal analysis according to baseline (2009 and/or 2010) *Aspergillus* colonization and ABPA status. Linear mixed effect models (ppFEV1, pBMI) and Cox's proportional hazards models (time to event) showing multivariable analysis after adjustment for known confounders*

**Clinical outcome**	***Aspergillus* colonization (n=263) versus no-*Aspergillus* (n=1412)**	**ABPA excluded: *Aspergillus* colonization (n=183) versus no-*Aspergillus* (n=1232)**	**ABPA (n=260) versus no-ABPA (n= 1415)**
**$ppFEV_1$** Between group mean difference (95% CI)	0.008 (-0.2 to 0.2) p=0.94 (n=1478)**	0.02 (-0.2 to 0.2) p=0.87 (n=1239)	-0.3 (-0.5 to -0.1) p=0.003 (n=1478)
**pBMI** Between group mean difference (95% CI)	-0.4 (-0.7 to -0.07) p=0.02 (n=1479)	-0.2 (-0.5 to 0.2) p=0.32 (n=1240)	-0.8 (-1.1 to - 0.5) p<0.0001 (n=1479)
**Time to death** HR (95% CI)	0.7 (0.4 to 1.3) p=0.26 (n=1485)	0.6 (0.3 to 1.2) p=0.17 (n=1247)	0.7 (0.4 to 1.2) p=0.20 (n=1485)
**Time to lung transplant** HR (95% CI)	1.3 (0.7 to 2.4) p=0.43 (n=1485)	0.9 (0.4 to 2.1) p=0.82 (n=1247)	1.1 (0.5 to 2.1) p=0.86 (n=1485)

$ppFEV_1$= percentage predicted forced expiratory volume in 1 second. pBMI= percentile body mass index. RC= Regression Coefficient, HR= Hazards Ratio, with 95% confidence interval in brackets. * Adjusted for known confounders: baseline age, sex, *CFTR* genotype, *P. aeruginosa* co-infection at baseline, nontuberculous mycobacteria at baseline, ppFEV1 (where not outcome), pBMI (where not outcome), ABPA (*Aspergillus* colonization versus no-*Aspergillus* colonization only), and CF related diabetes treatment at baseline. Patients with previous lung transplant excluded from clinical outcomes ppFEV1 and pBMI. ** Numbers in brackets are number of patients cited in the analyses.

Supplemental table S2: Longitudinal childhood sub-group analysis (including only those aged 8 to 11 years old at baseline) with time varying *Aspergillus* and ABPA status at annual review: *Aspergillus* colonization (≥1 positive respiratory culture in preceding year) and ABPA status (≥1 episode of ABPA in preceding year) at annual review. Mixed effect regression models* showing multivariable analysis after adjustment for known confounders**

**Clinical outcome**	***Aspergillus* colonization versus no-*Aspergillus* (n=733)**	**ABPA excluded (same year): *Aspergillus* colonization versus no-*Aspergillus* (n=733)**	**ABPA versus no-ABPA (n=733)**
**ppFEV$_1$** Between group mean difference (95% CI),	0.06 (-0.2 to 0.3)<br>p=0.59<br>(n=725)***	-0.1 (-0.3 to 0.2)<br>p=0.55<br>(n=714)	-0.5 (-0.8 to -0.3)<br>p<0.00001<br>(n=725)
**pBMI**Between group mean difference (95% CI)	-0.7 (-1.1 to -0.4)<br>p<0.00001<br>(n=725)	-0.7 (-1.0 to -0.3)<br>p=0.0009<br>(n=714)	-1.1 (-1.5 to -0.8)<br>p<0.00001<br>(n=725)
**IV antibiotic days** (during 12 months prior to annual review) OR (95% CI)	1.4 (0.7 to 2.8)<br>p=0.94<br>(n=725)	1.7 (0.8 to 3.9)<br>p=0.92<br>(n=713)	2.8 (1.7 to 4.6)<br>p=0.38<br>(n=725)
**Lung transplant** (in 12 months prior to annual review) OR (95% CI)	1.0 (0.2 to 5.3)<br>p=0.98<br>(n=661)	1.0 (0.2 to 5.3)<br>p=0.98<br>(n=661)	No result<br>(only 4 of childhood ABPA had Tx-lung)<br>(n=661)
**Death** (in 12 months prior to annual review) OR (95% CI)	0.5 (0.2 to 1.9)<br>p=0.33<br>(n=723)	0.5 (0.1 to 2.3)<br>p=0.36<br>(n=709)	2.1 (0.8 to 5.5)<br>p=0.13<br>(n=723)

ppFEV$_1$= percentage predicted forced expiratory volume in 1 second. pBMI = percentile body mass index. OR= Odds Ratio, with 95% confidence interval in brackets. *mixed effect regression models (linear mixed effect models (ppFEV1, pBMI); multilevel mixed effect negative binomial models (IV antibiotic days); and complementary log-log regression model (death and lung transplant). **Adjusted for known confounders: age, sex, *CFTR* genotype at baseline; *P. aeruginosa* co-infection, ppFEV1 (where not outcome), pBMI (where not outcome), and ABPA (column 1 only) in the same year. *** Numbers in brackets are number of patients cited in the analyses.

Supplemental table S3: Longitudinal adolescent sub-group analysis (including only those aged 12 to 17 years old at baseline) with time varying *Aspergillus* colonization and ABPA statusat annual review: *Aspergillus* colonization (≥1 positive respiratory culture in preceding year) and ABPA status (≥1 episode of ABPA in preceding year) at annual review. Mixed effect regression models* showing multivariable analysis after adjustment for known confounders**

Clinical outcome	*Aspergillus* colonization versus no-*Aspergillus* (n=942)	ABPA excluded (same year): *Aspergillus* colonization versus no-*Aspergillus* (n=942)	ABPA versus non-ABPA (n=942)
**$ppFEV_1$**<br>Between group mean difference (95% CI)	-0.1 (-0.3 to 0.2)<br>p=0.60<br>(n=925)***	-0.1 (-0.4 to 0.2)<br>p=0.58<br>(n=897)	-0.3 (-0.6 to -0.1)<br>p=0.01<br>(n=925)
**pBMI**<br>Between group mean difference (95% CI)	-0.5 (-0.8 to -0.08)<br>p=0.02<br>(n=925)	-0.3 (-0.7 to 0.2)<br>p=0.22<br>(n=897)	-0.6 (-0.9 to -0.2)<br>p=0.004<br>(n=925)
**IV antibiotic days**<br>(during 12 months prior to annual review)<br>OR (95% CI)	1.2 (0.7 to 1.9)<br>p=0.75<br>(n=925)	1.1 (0.6 to 2.0)<br>p=0.65<br>(n=896)	1.8 (1.2 to 2.6)<br>p=0.93<br>(n=925)
**Lung transplant**<br>(in 12 months prior to annual review)<br>OR (95% CI)	0.7 (0.3 to 1.9)<br>p=0.52<br>(n=923)	0.8 (0.3 to 2.0)<br>p=0.61<br>(n=895)	0.4 (0.08 to 1.5)<br>p=0.15<br>(n=923)
**Death**<br>(in 12 months prior to annual review)<br>OR (95% CI)	1.1 (0.5 to 2.2)<br>p=0.84<br>(n=858)	1.0 (0.4 to 2.2)<br>p=0.93<br>(n=821)	1.2 (0.6 to 2.5)<br>p=0.61<br>(n=858)

$ppFEV_1$= percentage predicted forced expiratory volume in 1 second. pBMI = percentile body mass index. , OR = Odds Ratio, with 95% confidence interval in brackets. *mixed effect regression models (linear mixed effect models (ppFEV1, pBMI); multilevel mixed effect negative binomial models (IV antibiotic days); and complementary log-log regression model (death and lung transplant). **Adjusted for known confounders: age, sex, *CFTR* genotype at baseline; *P. aeruginosa* co-infection, ppFEV1 (where not outcome), pBMI (where not outcome), and ABPA (column 1 only) in the same year. *** Numbers in brackets are number of patients cited in the analyses.

Supplemental table S4: Longitudinal sensitivity analysis on complete cases only with time varying *Aspergillus* colonization and ABPA status at annual review: *Aspergillus* colonization (≥1 positive respiratory culture in preceding year) and ABPA status (≥1 episode of ABPA in preceding year) at annual review. Mixed effect regression models* showing multivariable analysis after adjustment for known confounders**

**Clinical outcome**	***Aspergillus* colonization versus no-*Aspergillus* (n=1675)**	**ABPA excluded (same year): *Aspergillus* colonization versus no-*Aspergillus* (n=1675)**	**ABPA versus no-ABPA (n=1675)**
**ppFEV$_1$** Between group mean difference (95% CI)	-0.01 (-0.2 to 0.2) p=0.90 (n=1650)***	-0.1 (-0.3 to 0.1) p=0.34 (n=1609)	-0.4 (-0.6 to -0.3) p<0.00001 (n=1650)
**pBMI** Between group mean difference (95% CI)	-0.6 (-0.8 to -0.3) p<0.00001 (n=1650)	-0.4 (-0.7 to -0.1) p=0.005 (n=1609)	-0.8 (-1.1 to -0.5) p<0.00001 (n=1650)
**IV antibiotic days** (during 12 months prior to annual review) OR (95% CI)	1.3 (0.9 to 1.9) p=0.86 (n=1650)	1.3 (0.8 to 2.2) p=0.93 (n=1609)	2.2 (1.6 to 3.0) p=0.67 (n=1650)
**Lung transplant** (in 12 months prior to annual review) OR (95% CI)	0.7 (0.3 to 1.5) p=0.34 (n=1648)	0.7 (0.3 to 1.6) p=0.43 (n=1648)	0.2 (0.06 to 1.0) p=0.05 (n=1648)
**Death** (in 12 months prior to annual review) OR (95% CI)	0.9 (0.5 to 1.6) p=0.64 (n=1641)	0.8 (0.4 to 1.6) p=0.51 (n=1584)	1.3 (0.7 to 2.4) p=0.36 (n=1641)

ppFEV$_1$= percentage predicted forced expiratory volume in 1 second. pBMI = percentile body mass index. IV = intravenous. OR = Odds Ratio, with 95% confidence interval in brackets. *mixed effect regression models (linear mixed effect models (ppFEV1, pBMI); multilevel mixed effect negative binomial models (IV antibiotic days); and complementary log-log regression model (death and lung transplant). **Adjusted for known confounders: age, sex, *CFTR* genotype at baseline; *P. aeruginosa* co-infection, ppFEV1 (where not outcome), pBMI (where not outcome), and ABPA (column 1 only) in the same year. *** Numbers in brackets are number of patients cited in the analyses.

Supplemental table S5: Proportion of patients on CFTR modulator therapy over the period of study (2009-2019) according to time-varying *Aspergillus* colonization and ABPA status at annual review: *Aspergillus* colonization (≥1 positive respiratory culture in preceding year) and ABPA status (≥1 episode of ABPA in preceding year) at annual review

	Aspergillus* colonization versus no-*Aspergillus	**ABPA excluded (same year): *Aspergillus* colonisation versus no-*Aspergillus***	**ABPA versus no-ABPA**
**Proportion of patients on CFTR modulator therapy**	3.7% vs 3.6%<br>p=0.87	3.2% vs 3.3%<br>p=0.74	2.9% vs 3.3%<br>p=0.27

CFTR= cystic fibrosis transmembrane conductance regulator. P-value calculated with Chi-squared test.
